# Adding Early Postnatal Parameters of Ventilation to Prognostic Models for Pulmonary Outcome in Very Preterm Infants

**DOI:** 10.1002/ppul.71335

**Published:** 2025-11-12

**Authors:** Birte Staude, Eva‐Maria Mair, Maria Zernickel, Antje Westhoff, Rahel Schuler, Frank Oehmke, Harald Ehrhardt

**Affiliations:** ^1^ Division of Neonatology and Pediatric Intensive Care Medicine, Department of Pediatrics and Adolescent Medicine University Medical Center Ulm Ulm Germany; ^2^ Department of General Pediatrics & Neonatology Justus Liebig University and Universities of Giessen and Marburg Lung Center Giessen Germany; ^3^ Department of Pediatrics and Adolescent Medicine, Centre for Pediatric Clinical Studies University Medical Center Ulm Ulm Germany; ^4^ Department of Gynecology and Obstetrics Justus Liebig University of Giessen Giessen Germany

**Keywords:** bronchopulmonary dysplasia, mean airway pressure, preterm, prognostic model, respiratory distress syndrome, respiratory support, score, supplemental oxygen

## Abstract

**Aim:**

To compare discrimination and calibration of prognostic models for pulmonary outcomes in very preterm (VPT) infants born < 32 weeks’ gestation when including the mean airway pressure (MAP), the fraction of supplemental oxygen (FiO_2_) and the respiratory severity score (RSS) reflecting parameters of ventilation and oxygenation during the first 24 and 72 h of life.

**Methods:**

In this retrospective single center study of 168 VPT infants, the mean airway pressure (MAP), the fraction of supplemental oxygen (FiO_2_) or RSS (considering MAP and FiO_2_) were added to a baseline model of clinical risk factors to assess the improvements for prediction of bronchopulmonary dysplasia (BPD).

**Results:**

The baseline model demonstrated good calibration (slope 1.02) and discrimination (AUC 0.85) for overall BPD (BPD28), and adding any of the parameters of ventilation resulted only in slight improvement in discrimination (AUC 0.86). For moderate/severe BPD (BPD36), overprediction in the lower extremes and underprediction in the upper extremes became evident for the baseline model. While adding MAP rendered optimal specificity (81%), sensitivity (91%) was highest for FiO_2_. MAP was substantially better at improving calibration for BPD36 (slope 0.98) than FiO_2_ (slope 0.87). Using RSS and expanding the models to the first 72 h of life did not result in any improvements.

**Conclusion:**

Adding parameters of ventilation and oxygenation improves baseline models to predict the risk of BPD28 and BPD36 early after birth. Particularly, our data encourage considering MAP as potential predictor in the development of future risk models to improve the prediction accuracy and to solidify early treatment decisions intended to prevent BPD.

**Abbreviations**
VPT: very preterm
FiO_2_
: fraction of inspired oxygen
RSS: respiratory severity score
BPD: bronchopulmonary dysplasia
RDS: respiratory distress syndrome
NICU: neonatal intensive care unit
PNS: postnatal corticosteroids
ANS: antenatal corticosteroids
MAP: mean airway pressure
PIP: peak inspiratory pressure
IQR: interquartile range
ROC: receiver operating characteristic
AUC: area under the curve
NIV: noninvasive ventilation
NIPPV: nasal intermittent positive pressure ventilation
SGA: small for gestational age
LISA: less invasive surfactant application
ROP: retinopathy of prematurity
IVH: intraventricular hemorrhage
PVL: periventricular leukomalacia
NEC: necrotizing enterocolitis
PDA: patent ductus arteriosus

## 1 Introduction

Very preterm (VPT) infants born before 32 weeks’ gestation are at high risk for respiratory distress syndrome (RDS) due to impaired gas exchange of the immature lung and lack of surfactant [1]. Supplemental oxygen and mechanical ventilation necessary for the treatment of RDS are main contributors to the development of bronchopulmonary dysplasia (BPD), the chronic lung disease of premature infants leading to long‐term consequences for respiratory health and neurodevelopment [1]. Postnatal corticosteroids (PNS) have been proven to be efficacious in preventing BPD [2]. However, drawbacks like a potential negative impact of PNS on BPD and neurodevelopmental impairment in populations with a low baseline risk for BPD prohibit a wide use [3, 4, 5]. Hence, an early identification of infants at high risk of BPD to target PNS and other therapies intended to prevent BPD is important [6].

While radiologic grading of RDS has been clinical standard for decades, its prediction accuracy for BPD is limited [7]. Especially since the provision of surfactant in the delivery room and the use of noninvasive ventilation (NIV) has become more common, the validity of radiologic grading to determine severity of RDS has come into question, as x‐rays are often only obtained after initial surfactant administration [7]. Several scores based on initial ventilation and oxygenation parameters to determine RDS severity have been developed. The scores including FiO_2_ like the peripheral oxygen saturation (SpO_2_)/fraction of inspired oxygen (FiO_2_) ratio probably reflect the simplest approach as they only rely on the parameters of oxygenation [8]. For describing the pressure transduction to the lung during different modes of mechanical ventilation (MV), the mean airway pressure (MAP) has evolved as standard as it is independent of the mode and reflects peak inspiratory pressure and ventilator frequency [9, 10]. The more sophisticated scores of oxygen index (OI, MAP*FiO_2_*100/partial pressure of oxygen) and oxygen saturation index (OSI, MAP*FiO_2_/SpO_2_) and respiratory severity score (RSS, MAP*FiO_2_) consider FiO_2_ and MAP, and thereby respect lung aeration and oxygenation [8, 11]. The value of including these scores in the prediction of BPD has been assessed rarely before and correlations with BPD were described only for their calculation beyond the second week of life [12, 13].

Multiple clinical prediction models for BPD have been suggested but prediction accuracy of models in the first week of life is limited [14]. Furthermore, most of the studies have methodological limitations and do not describe calibration of their model [6, 14]. Usually included predictors include antenatal corticosteroids (ANS) which constitute a major determinant for less severe RDS and improvements in gas exchange after birth but cannot reduce the risk of BPD, as well as non‐modifiable factors including birth weight and sex, which are of much higher relevance [15]. Many models include the fraction of inspired oxygen and mode of respiratory support but only few consider MAP [14].

When assessing a predictive model, it is crucial to not only asses how well the model can differentiate between infants who do or do not develop the disease (discrimination, usually measured by receiver operating characteristics and area under the curve (AUC)) but also how well the model predicts the specific probabilities (calibration). Calibration can be assessed in the large (e.g., by calibration slopes) as well as locally (by calibration plots) and is especially relevant when clinical decisions are meant to be based on predicted probabilities [16].

The aim of this retrospective cohort study was to assess changes in discrimination and calibration of prediction models for BPD when adding the different parameters of oxygenation and ventilation (i.e. MAP, the FiO2 or the RSS) to a base model while including invasively and non‐invasively ventilated infants. Special focus was set on parameters available early after birth.

## 2 Material and Methods

### 2.1 Study Design, Setting and Ethics Vote

This single‐center retrospective cohort study consecutively included VPT infants < 32 weeks´ gestation born between 2013 and 2021 admitted to the perinatal center of the Justus‐Liebig‐University Giessen. The inclusion criterion was based on that of other studies on the topic like the EPICE cohort [17]. Exclusion criteria comprised major congenital malformations particularly of the lung and cardiovascular system, severe syndromal diseases and death before 36 weeks´ gestation as determination of BPD disease severity is executed at this timepoint. Furthermore, patients with missing outcome data due to transfer to another neonatal intensive care unit (NICU) were excluded. All patient data were derived from the hospitals´ electronic data management system and the paper file records. The study was conducted following the rules of the Declaration of Helsinki of 1975, revised in 2013, and was approved by the ethics committees of the Justus‐Liebig‐University Giessen (Az 98/14). Parental consent was not necessary due to the retrospective analysis. Reporting follows the TRIPOD‐AI statement [18].

### 2.2 Outcome

Predefined outcomes were BPD defined as the need for supplemental oxygen for > 28 days according to the National Institute of Child Health and Human Development (NICHD) 2001 definition (BPD28), as well as the need for oxygen or ventilatory support at 36 weeks post menstrual age (BPD36) [19]. Furthermore, we evaluated the latest NICDH 2019 definition on our sample [20]. Infants received all common modes of invasive and noninvasive respiratory support including synchronized intermittent ventilation, high frequency oscillation, nasal intermittent positive pressure ventilation or nasal continuous positive airway pressure (nCPAP) and highflow therapy as prescribed by the attending physician. During nCPAP therapy, the positive endexpiratory pressure was used to estimate pressure transmission. The positive end‐expiratory pressure level during highflow therapy and the fraction of oxygen supplied via nasal cannula were estimated by converters as published recently [21, 22]. Highflow was counted as continuous positive airway pressure (CPAP) when the level of positive endexpiratory pressure (PEEP) was 3cmH_2_O or higher [23].

### 2.3 Predictors

Included predictors were selected according to their clinical relevance and previous published BPD risk prediction models. We added ANS, as appropriate timing of ANS alleviates the respiratory course after birth. To allow early prediction, we only included predictors readily available on the first day of life. Gestational age and small for gestational age status were not included because of the strong collinearity with birth weight.

From the parameters of ventilation and oxygenation, MAP, FiO_2_ or RSS were included individually. MAP was selected as parameter of ventilation for its best applicability to describe the pressure transduction to the lung during different modes of ventilation and a correction factor of ‐2.6cmH_2_O was applied for NIV [24]. When MAP was not available during NIV, it was calculated as described before (respiratory rate * 0.3/60 * (PIP‐PEEP) + PEEP) [25]. Correctness of calculations was verified by Bland Altman plots in patients with noninvasive MAP readings of the ventilator available. For infants on CPAP, PEEP was considered as MAP and corrected by ‐2.6cmH_2_O as well. For infants on highflow, pharyngeal pressure was calculated as published (PEEP = 2.6 + (0,8 x flow[l/min]) – (1.4 x weight[kg])) [22]. The RSS was calculated as published (RSS = MAP * FiO_2_) [8]. FiO_2_ and MAP values were used from the highest requirement and the most invasive mode of respiratory support during the first 24 or 72 h of life documented beyond the initial stabilization in the delivery room. The timepoints were chosen to focus on the possibility of an early prediction and to investigate if expanding the timeframe to 72 h is beneficial.

Definitions of further items included in descriptive statistics but not in the prognostic models can be found in the online supporting material.

### 2.4 Statistical Methods

The statistical analyses were performed using R, version 4.4.2 (R Foundation for Statistical Computing, Vienna, Austria) [26]. The demographics and outcomes are shown as medians and interquartile ranges. For counted data, the absolute and relative frequencies of the parameters are presented. Statistical comparisons were carried out using the Wilcoxon rank‐sum test for metric data and Pearson's Chi‐square with continuity correction/Fisher's exact test for categorical variables. Statistical significance was accepted with a *p*‐value < 0.05.

### 2.5 Model Development

For BPD28 and BPD36 a base logistic regression model, consisting of birthweight, sex and timing of antenatal corticosteroid administration was built respectively. Birthweight was included in units of 100 g to allow for easier interpretation of odds ratios and handled as linear predictor. Sex was included as dichotomous, timing of antenatal corticosteroids as factor with three levels according to the timing of their last application (none or < 24 h before birth, 24 h to 7 days before birth, > 7 days before birth). MAP was included in cmH_2_O, FiO_2_ was transferred to percent to allow for interpretation of odds ratios. RSS was kept as is. All three parameters of ventilation were considered as linear, polynomials and logarithmic transformation were considered but did not yield better results.

For BPD28 and BPD36, three models were built for day 1 and day 3 of life respectively by including either FiO_2_, MAP or RSS to the baseline model. Within every modelbuilding process, the sample was bootstrapped 1000 times. The model was then fitted on the bootstrap sample and validated on the bootstrap and original sample [27]. Average optimism was calculated, the coefficients of the final model were shrunk according to the optimism corrected calibration slope and the intercept was recalculated [27].

### 2.6 Model Evaluation

Internal validation was assessed by receiver operating characteristic (ROC) curves and c‐statistic as well as calibration plots and slopes using R packages pROC [28] and CalibrationCurves [29]. Measures of accuracy were assessed for each model for the threshold with the highest Youden‐Index [30].

### 2.7 Sample Size

Overall, 168 infants were available for the analyses spanning a 9‐year period without fundamental changes in the respiratory management. Due to the retrospective character of the cohort, no a priori sample size calculation was possible. Sample size calculations based on the final models were conducted using the pmsampsize [31] package in R.

### 2.8 Missing Data

Participants with missing outcome due to death or transfer before 36 weeks were excluded. There were no missing data for the included predictors, except for measured MAP during NIV (61.4%), which was calculated as mentioned above. Bland Altman Plots did confirm the validity of this calculation in our dataset with only slight deviation of the median of ‐0.5 cmH_2_O (Supporting Information S1: Figure 1).

## 3 Results

From the total sample of 168 infants, 97 infants (57.7%) fulfilled the criterion BPD28 and of these, 32 (19.0%) BPD36 according to the NICHD 2001 definition. BPD36 cases were identical to cases with BPD when applying the new NICHD 2019 definition on our cohort.

### 3.1 Maternal and Neonatal Baseline Characteristics and BPD

The maternal and neonatal baseline characteristics divided by outcome are summarized in Table 1 for BPD36 and Supporting Information S1: Table 1 for BPD28. There were no significant differences in maternal baseline characteristics including maternal age, number of pregnancy and parity, maternal nicotine consumption, type of pregnancy, mode of delivery and cause of preterm delivery. Only for ANS, preterm infants within both BPD categories received ANS more frequently within the optimal time window. As can be expected from the published literature, infants with BPD had a significantly lower birth weight compared to the no BPD category (median 840 g vs. 1300 g for BPD28, 710 g vs. 990 g for BPD36). Comparable differences were observed for gestational age while there were no differences in sex distribution, Apgar scores and umbilical cord artery pH. Infants with BPD experienced more frequently a severe non‐pulmonary outcome including patent ductus arteriosus (PDA), PDA therapy, retinopathy of prematurity (ROP) and ROP therapy as well as late onset infection (LOI), while for intraventricular hemorrhage (IVH) and periventricular leukomalacia (PVL) statistically significant differences were only detected for BPD36.

**TABLE 1:** Maternal and neonatal baseline characteristics by BPD36.

	Overall	no BPD36	BPD36	*p* value
N (%)	168 (100.0%)	136 (81.0%)	32 (19.0%)	
**Neonatal baseline characteristics**				
**Birthweight [g]**				
Median [IQR]	970.0 [758.8, 1285.0]	990.0 [850.0, 1350.0]	710.0 [577.5, 852.5]	< 0.001
**Gestation [weeks + days]**				
Median [IQR]	28 + 1 [26 + 3, 30 + 0]	28 + 6 [27 + 2, 30 + 1]	25 + 5 [24 + 5, 26 + 4]	< 0.001
**Sex**				
female	92 (54.8%)	75 (55.1%)	17 (53.1%)	0.99
male	76 (45.2%)	61 (44.9%)	15 (46.9%)	
**Apgar 1‘**				
Median [IQR]	8.0 [7.0, 8.0]	8.0 [7.0, 8.0]	8.0 [7.0, 8.0]	0.7
**Apgar 5‘**				
Median [IQR]	9.0 [8.0, 9.0]	9.0 [8.0, 9.0]	9.0 [8.0, 9.0]	0.13
**Apgar 10‘**				
Median [IQR]	10.0 [9.0, 10.0]	10.0 [9.0, 10.0]	9.5 [9.0, 10.0]	0.41
**UA‐pH**				
Median [IQR]	7.3 [7.2, 7.4]	7.3 [7.2, 7.4]	7.3 [7.3, 7.4]	0.21
Missing (%)	22 (13.1%)	32 (23.5%)	8 (25.0%)	
**X‐ray within first 72 h ‐ RDS grade**				
1	34 (20.2%)	27 (19.9%)	7 (21.9%)	0.38
2	52 (31.0%)	41 (30.1%)	11 (34.4%)	
3	35 (20.8%)	23 (16.9%)	12 (37.5%)	
4	8 (4.8%)	7 (5.1%)	1 (3.1%)	
Missing (%)	39 (23.2%)	38 (27.9%)	1 (3.1%)	
**Surfactant**				
Yes	107 (63.7%)	78 (57.4%)	29 (90.6%)	< 0.001
**Surfactant doses within 72 h**				
Median [IQR]	1.0 [0.0, 1.0]	1.0 [0.0, 1.0]	1.0 [1.0, 1.2]	< 0.001
**Maternal baseline characteristics**				
**Maternal Age**				
Median [IQR]	31.0 [27.0, 36.0]	31.0 [27.8, 36.0]	31.0 [26.8, 35.0]	0.73
**Pregnancy**				
Median [IQR]	1.0 [1.0, 3.0]	1.0 [1.0, 3.0]	1.0 [1.0, 2.2]	0.94
**Parity**				
Median [IQR]	1.0 [1.0, 2.0]	1.0 [1.0, 2.0]	1.0 [1.0, 1.0]	0.34
**Mode of delivery**				
C‐section	161 (95.8%)	130 (95.6%)	31 (96.9%)	> 0.999
Spontaneous	7 (4.2%)	6 (4.4%)	1 (3.1%)	
**Type of pregnancy**				
singleton	104 (61.9%)	85 (62.5%)	19 (59.4%)	0.9
multiple	64 (38.1%)	51 (37.5%)	13 (40.6%)	
**Nicotine**				
yes	26 (15.5%)	21 (15.4%)	5 (15.6%)	> 0.999
Missing (%)	3 (1.8%)	3 (2.2%)	0 (0.0%)	
**ANS**				
none/< 24 h	38 (22.6%)	31 (22.8%)	7 (21.9%)	0.04
> 24 h/< 7 d	86 (51.2%)	64 (47.1%)	22 (68.8%)	
> 7 d	44 (26.2%)	41 (30.1%)	3 (9.4%)	
**Cause for preterm delivery**				
AIS	76 (45.2%)	62 (45.6%)	14 (43.8%)	0.88
Preeclampsia/HELLP	33 (19.6%)	28 (20.6%)	5 (15.6%)	
IUGR	23 (13.7%)	18 (13.2%)	5 (15.6%)	
Other	36 (21.4%)	28 (20.6%)	8 (25.0%)	
**Morbidities of prematurity**				
**PDA**				
no	37 (22.0%)	30 (22.1%)	7 (21.9%)	0.02
PDA	46 (27.4%)	25 (18.4%)	21 (65.6%)	
Missing (%)	85 (50.6%)	81 (59.6%)	4 (12.5%)	
**PDA therapy (any)**				
yes	9 (5.4%)	0 (0.0%)	9 (28.1%)	< 0.001
**ROP**				
no	107 (63.7%)	103 (75.7%)	4 (12.5%)	< 0.001
yes	61 (36.3%)	33 (24.3%)	28 (87.5%)	
**ROP grade**				
no ROP	107 (63.7%)	103 (75.7%)	4 (12.5%)	< 0.001
1	29 (17.3%)	21 (15.4%)	8 (25.0%)	
2	13 (7.7%)	8 (5.9%)	5 (15.6%)	
≥ 3 including APROP	19 (11.3%)	4 (2.9%)	15 (46.9%)	
**ROP therapy (any)**				
ROP with therapy	10 (6.0%)	1 (0.7%)	9 (28.1%)	< 0.001
**PVL**				
PVL	3 (1.8%)	0 (0.0%)	3 (9.4%)	0.004
**IVH**				
IVH	10 (6.0%)	5 (3.7%)	5 (15.6%)	0.03
**IVH grade**				
0	158 (94.0%)	131 (96.3%)	27 (84.4%)	0.005
1	4 (2.4%)	3 (2.2%)	1 (3.1%)	
2	5 (3.0%)	1 (0.7%)	4 (12.5%)	
3	1 (0.6%)	1 (0.7%)	0 (0.0%)	
4	0 (0.0%)	0 (0.0%)	0 (0.0%)	
**Pneumothorax**				
yes	3 (1.8%)	2 (1.5%)	1 (3.1%)	> 0.999
**Infection**				
LOI	21 (12.5%)	8 (5.9%)	13 (40.6%)	< 0.001
**Duration of invasive ventilation and noninvasive ventilatory support**				
**Total time respiratory support (MV** + **NIV) [days]**				
Median [IQR]	33.0 [10.0, 54.0]	28.0 [8.0, 43.0]	72.0 [63.0, 96.0]	< 0.001
Missing (%)	3 (1.8%)	0 (0.0%)	3 (9.4%)	
**Invasive ventilation [days]**				
Median [IQR]	0.0 [0.0, 1.0]	0.0 [0.0, 0.0]	1.0 [0.0, 9.5]	< 0.001

Abbreviations: ANS, antenatal corticosteroids; BPD, bronchopulmonary dysplasia; IVH, intraventricular hemorrhage; LOI, late onset infection; PDA, patent ductus arteriosus; PVL, periventricular leukomalacia; ROP, retinopathy of prematurity; UA‐pH, umbilical artery pH.

### 3.2 Respiratory Course After Birth Segregated by the BPD Definition

Next, we report the respiratory course during the first 24 and 72 h of life. Infants with BPD28 and BPD36 respectively, more frequently received surfactant and were intubated and mechanically ventilated, although radiologic RDS severity stages did not differ significantly between the groups (Tables 1, 2, Supporting Information S1: Tables 1–2). In line, the duration of mechanical ventilation and total respiratory support was significantly longer. There were no differences in the rates of pneumothorax. When focussing on the ventilator settings, there were no differences for the PEEP, MAP and PIP when infants were segregated for MV and NIV as highest respiratory support measure during their first 24 and 72 h of life, except for MAP under NIV in the BPD28 definition and when calculated MAP was included for participants with missing MAP. Along with higher rates of intubation in the BPD28 and BPD36 category, MAP was significantly higher in these categories after correction for variations in pressure transduction to the lung for MV and NIV. FiO_2_ was as well significantly higher in BPD28 and BPD36 categories (Table 2 and Supporting Information S1: Table 2).

**TABLE 2:** Ventilatory parameters within the first 24 and 72 h of life by BPD36.

	Overall	no BPD36	BPD36	*p* value
N (%)	168 (100.0%)	136 (81.0%)	32 (19.0%)	
**Ventilation within first 24 h**				
**Most invasive mode of respiratory support within 24 h**				< 0.001
HFNC	3 (1.8%)	3 (2.2%)	0 (0.0%)	
CPAP	27 (16.1%)	27 (19.9%)	0 (0.0%)	
NIPPV	91 (54.2%)	78 (57.4%)	13 (40.6%)	
IPPV	45 (26.8%)	28 (20.6%)	17 (53.1%)	
HFO	2 (1.2%)	0 (0.0%)	2 (6.2%)	
**MAP**_**NIV**_ **(measured, not including calculated)**				
Median [IQR]	8.7 [8.0, 9.2]	8.6 [8.0, 9.3]	9.0 [7.9, 9.1]	0.97
Missing (%)^a^	54 (59.3%)	48 (61.53%)	6 (46.15%)	
**MAP**_**C‐NIV**_ **(MAP**_**NIV**_ **including calculated + correction for NIV)†**				
Median [IQR]	6.1 [4.6, 7.0]	5.9 [4.4, 7.0]	6.5 [6.1, 7.4]	0.054
**PIP**_**MV**_				
Median [IQR]	16.0 [14.0, 20.0]	15.5 [14.0, 20.0]	16.0 [14.0, 19.0]	0.93
**PEEP**_**MV**_				
Median [IQR]	6.0 [5.4, 6.2]	6.0 [5.4, 6.2]	6.0 [5.8, 6.2]	0.61
**MAP**_**MV**_				
Median [IQR]	8.6 [7.6, 9.7]	8.4 [7.7, 9.3]	9.0 [7.5, 9.8]	0.59
**MAP**_**C‐TP**_ **(including correction for NIV)†**				
Median [IQR]	6.7 [5.3, 7.8]	6.6 [4.7, 7.5]	7.6 [6.5, 9.1]	< 0.001
**Ventilation within first 72 h**				
**Most invasive mode of respiratory support within 72 h**				< 0.001
HFNC	3 (1.8%)	3 (2.2%)	0 (0.0%)	
CPAP	24 (14.3%)	24 (17.6%)	0 (0.0%)	
NIPPV	89 (53.0%)	78 (57.4%)	11 (34.4%)	
IPPV	49 (29.2%)	31 (22.8%)	18 (56.3%)	
HFO	3 (1.8%)	0 (0.0%)	3 (9.4%)	
**FiO**_**2**_				
Median [IQR]	0.3 [0.3, 0.5]	0.3 [0.2, 0.5]	0.4 [0.4, 0.6]	< 0.001
**PIP**_**NIV**_				
Median [IQR]	15.1 [15.0, 17.0]	15.2 [15.0, 17.0]	15.0 [15.0, 17.0]	0.65
**PEEP**_**NIV**_				
Median [IQR]	7.0 [6.8, 8.0]	7.0 [6.6, 8.0]	7.0 [7.0, 7.0]	0.33
**MAP**_**NIV**_ **(measured, not including calculated)**				
Median [IQR]	8.7 [8.0, 9.1]	8.7 [8.3, 9.2]	8.5 [7.8, 9.1]	0.6
Missing (%)^a^	54 (61.4%)	49 (63.6%)	5 (45.5%)	
**MAP**_**C‐NIV**_ **(MAP**_**NIV**_ **including calculated + corrected for NIV)**				
Median [IQR]	6.5 [5.3, 9.3]	6.5 [5.2, 9.2]	6.5 [5.9, 9.8]	
**PIP**_**MV**_				
Median [IQR]	16.0 [15.0, 20.0]	16.0 [14.9, 20.0]	16.5 [15.0, 21.0]	0.43
**PEEP**_**MV**_				
Median [IQR]	6.0 [5.4, 6.3]	5.9 [5.3, 6.2]	6.1 [5.8, 6.8]	0.14
**MAP**_**MV**_				
Median [IQR]	8.7 [8.0, 9.1]	8.4 [7.7, 9.6]	9.4 [8.5, 11.0]	0.03
**MAP**_**C‐TP**_ **(including correction for NIV)**				
Median [IQR]	7.7 [5.9, 9.4]	7.2 [5.4, 9.2]	9.2 [7.5, 10.5]	< 0.001

Abbreviations: BPD, bronchopulmonary dysplasia; FiO2, fraction of inspired oxygen; MAP, mean airway pressure; MV, mechanical ventilation; NIV, noninvasive ventilation; PEEP, positive end‐expiratory pressure; PIP, positive inspiratory pressure; TP, total population

^a^ Missing data calculated as a proportion of those receiving NIV.

### 3.3 Adding Ventilation Parameters to Improve the Prediction Accuracy for BPD

As published in the literature, birth weight had the highest impact on the prediction of BPD for both definitions. Our baseline model demonstrated good calibration and discrimination for the prediction of BPD28. Addition of FiO_2_ or MAP did improve the accuracy from 0.76 to 0.79 and the AUC from 0.85 to 0.86. Sensitivity was highest when adding FiO_2_ (80%) while specificity was best when adding MAP (94%) (Supporting Information S1: Table 3, Supporting Information S1: Figure 2–3). For BPD36, the baseline model displayed overprediction in the lower extremes and underprediction in the upper extremes. Calibration was worse when adding FiO_2_ to the model but improved when adding MAP (Supporting Information S1: Figure 4–5, Table 3). As for BPD28, addition of FiO_2_ resulted in the best sensitivity (91%) while specificity was highest (81%) when adding MAP (Table 3). Addition of the RSS instead of FiO_2_ or MAP did not further improve the accuracy of the model (Table 3). Similarly, extension of the observation period to 72 h had no advantage compared to considering the first 24 h of life (Table 3). Full models are reported in the supplemental material (Supporting Information S1: Tables 4 and 5).

**TABLE 3:** Model discrimination for BPD36.

Model AUC threshold specificity sensitivity PPV NPV accuracy calibration slope								
Model	AUC	Threshold	Specificity	Sensitivity	PPV	NPV	Accuracy	Calibration slope
Base model	0.83	0.22	0.75	0.84	0.44	0.95	0.77	0.93
**First 24 h including:**								
FiO_2_	0.83	0.16	0.68	0.91	0.40	0.97	0.72	0.87
MAP	0.85	0.26	0.81	0.78	0.49	0.94	0.80	0.98
RSS	0.85	0.22	0.76	0.84	0.45	0.95	0.77	0.88
**First 72 h including:**								
FiO_2_	0.84	0.20	0.72	0.88	0.42	0.96	0.75	0.89
MAP	0.84	0.22	0.78	0.81	0.46	0.95	0.79	0.96
RSS	0.85	0.22	0.76	0.84	0.45	0.95	0.77	0.89

*Note:* Base model consists of birthweight, sex and antenatal corticosteroids. Full models are shown in Supporting Information S1: Tables 1 and 2.

## 4 Discussion

### 4.1 Principal Findings

It is of high clinical relevance to have objective parameters and scores at hand that enable the clinician to validly judge the individual patient´s risk of BPD as early as possible. Most studies to estimate the risk of BPD relied on baseline items, partly combined with postnatal diagnoses and parameters of oxygenation. The results of this study are encouraging as they indicate that adding both parameters of ventilation and oxygenation obtained shortly after birth are suited to improve the prediction model for BPD outcomes compared to using exclusively baseline characteristics. The particular advantage of our model approach is that it is applicable to all VPT infants at risk as it uses correction of MAP for infants on NIV. It thereby respects the growing trend to apply surfactant less invasively to VPT infants and to avoid mechanical ventilation whenever possible due to the injurious harms to the immature lung [1, 32, 33, 34]. We preferred studying BPD as individual component instead of a composite outcome together with mortality as these two items may have differing associations and to provide granularity for the BPD outcome. And in our cohort most deaths occurred for non‐pulmonary causes. The equal accuracy of the models for the 24‐ and 72‐h observation period indicate that early postnatal judgment of the risk is feasible. When considering the calibration, which was missing in previously published models, MAP yielded better results in our data than FiO_2_. This should encourage to collect information on MAP, which is often disregarded, in future cohorts and to consider it as a predictor candidate in future model development and adjustment.

### 4.2 Putting the Results Into the Context of the Published Literature

Our approach indicates that expanding the clinical risk models from baseline factors like gestational age to the objective parameters of ventilation and oxygenation is promising, especially for BPD36. This is in line with the available literature on other research fields like sepsis assessment where objective clinical parameters are retrieved [35]. For BPD, most models rely on baseline risks and postnatal outcomes like a hemodynamically significant PDA that is hard to judge objectively during clinical routine and where experts in the field have divergent thoughts on which items are best suited to determine hemodynamic significance [36, 37, 38, 39]. Other studies indicate that early postnatal clinical assessment including the Apgar score as candidate are suited to improve the prediction accuracy [40]. But one has to keep in mind that Apgar scoring is subject to intra‐observer and inter‐observer variability and one latest publication indicated that local validation and accounting for country‐specific effects is indispensable [41]. The current version of the NICHD BPD risk calculator circumvents these limitations by relying on baseline characteristics, necrotizing enterocolitis (NEC) as a severe outcome occurring during the stay in the NICU, the mode of ventilation and FiO_2_ [6]. The NICHD model has an AUC of 0.67 in internal validation for day one of life [6]. Our model comprising three clinical baseline items and either the maximum FiO2 or the maximum MAP rendered an AUC of 0.85 in internal validation. We did not include chorioamnionitis, PDA estimate and postnatal infections as risk factors for BPD into our model as these items are usually not available shortly after delivery to allow early risk estimation. In comparison to the NICHD model, we did not directly include the mode of respiratory support as an individual predictor, as the mode of respiratory support already contributes to the MAP and correction of MAP for noninvasive modes was incorporated. When considering our data, it might be worth to exploit the advantage of adding MAP to the already established NICHD model.

Early prediction of BPD is imperative for early treatment decisions to reach maximum efficacy and to avoid the potential harms of such treatments to infants without a major risk for BPD. The best example for this need is certainly postnatal corticosteroid therapy to prevent BPD. Based on the most recent advances, the advantage is only given for infants at high risk for BPD while those with a medium or low risk are particularly prone to the potential adverse effects on the psychomotor outcome [3]. In this respect, the current version of the NICHD BPD risk calculator, as most proposed model, has the disadvantage that prediction accuracy is suboptimal in the first week and adding or exchanging items could improve this shortcoming. It is reassuring that the studied models relying on 3 baseline risks and one additional parameter of ventilation prevailed specificity and prediction accuracy comparable to those of previous studies and even more detailed models [6, 14, 42, 43]. Lastly, it will be important to see if models are applicable as well to the other acute and longer‐term severe outcomes of prematurity including ROP and psychomotor impairment as pathomechanisms of disease origin have relevant homologies to that for BPD.

### 4.3 Strengths and Limitations of the Study

Our analysis has several strengths: First, it includes infants on NIV and MV and thereby is applicable to the total cohort of VPT infants. And we were able to include the old NICHD 2001 BPD definition and the latest 2019 version making it globally applicable. Second, it derives from a recent cohort with early application of less invasive surfactant administration (LISA) as standard of care and a high rate of successful stabilization on NIV (70.2%). Third, the parallel consideration of the first 24 and 72 h after delivery indicates that these predictors are reliable early on, while the prediction in the first weeks is one limitation of BPD prediction models [14]. Fourth, patient characteristics for important confounders for BPD including sex, cause of preterm delivery and maternal nicotine consumption during pregnancy did not differ between the groups what reduces the risk of missing an important predictor [15]. PDA was not included, as this information is not commonly available at the first day of life in our unit.

By using those ventilation modes with proven superiority like synchronized nasal intermittent positive pressure ventilation during NIV, state‐of‐the art respiratory management was secured on our cohort [44]. Due to the inclusion of easily available predictors, we did not have missing data, except for measured MAP, where we relied on an established calculation, excluding risk of bias due to missing data assumptions. We are not expecting problems with data acquisition during clinical application of a model including these predictors. Last, as we did not require informed parental consent for our retrospective analyses, we had no patient drop‐outs, reducing the risk of selection bias.

However, we need to acknowledge limitations of our study. First, due to the retrospective nature we integrated most items relevant to the topic into our analyses, but the number of included predictors was limited by the availability of data. Back calculation of the sample size revealed that our retrospective cohort was slightly too small and the negative results for the RSS should be considered with caution as it incorporates FiO_2_ and MAP and thereby is subject to higher imprecision. Considering the above‐mentioned predictors including one factor with three levels, the c‐statistic of 0.85 and a maximum shrinkage of 0.8 as well as the observed prevalences, a sample size of 237 would have been necessary for BPD28 and 377 for BPD36 for RSS. As an external validation cohort with MAP was not available, it will be imperative to validate the findings in independent cohorts. Second, our data apply to conventional modes of ventilation during NIV and MV as this was the primary ventilation mode in our NICU while high frequency oscillation was reserved to cases with severe respiratory failure. Therefore, it will be necessary to evaluate the findings in cohorts using the different modes of NIV and MV. But this pragmatic approach provides data from a real‐life scenario where it was up to the clinician´s discretion to select and change the mode of respiratory support and to manage respiratory stimulant therapy. However, due to the multifactorial aetiology of BPD it seems unlikely that this factor would reverse the overall results, especially since BPD rates are described to be constant across varying clinical practices for more than two decades [45, 46].

## 5 Conclusion

Overall, our results should encourage to focus research on BPD risk prediction models to the first day of life, including parameters of ventilation. MAP is easily available in the clinical setting without additional cost and, by correction for NIV, allows for the inclusion of MV and NIV in the same model. The calibration curves prevailed that inclusion of MAP was superior to FiO_2_ in our data. Our results are encouraging that improvement of early BPD risk prediction is possible with objective clinical parameters that can easily be executed under a protocol of minimal handling and without disturbing postnatal adaptation for example with echocardiographic evaluation.

## Author Contributions

**Birte Staude:** conceptualization, investigation, formal analysis, writing – original draft, writing – review and editing. **Eva‐Maria Mair:** investigation, writing – review and editing. **Maria Zernickel:** investigation, writing – review and editing. **Antje Westhoff:** investigation, writing – review and editing. **Rahel Schuler:** investigation, writing – review and editing. **Frank Oehmke:** investigation, writing – review and editing. **Harald Ehrhardt:** conceptualization, writing – original draft, writing – review and editing, supervision.

## Conflicts of Interest

The authors declare no conflicts of interest.

## Supporting information

250825Ventilation parameters after birth and BPD supplement.

## Data Availability

The data that support the findings of this study are available from the corresponding author upon reasonable request.
