# Supplementary material for: Adding Early Postnatal Parameters of Ventilation to Prognostic Models for Pulmonary Outcome in Very Preterm Infants
Source: Pediatr Pulmonol. 2025 Nov 12;60(11):e71335. doi: 10.1002/ppul.71335 (PMC12606693; doi:10.1002/ppul.71335)
Supplement: Supplementary file 1 — 250825Ventilation parameters after birth and BPD supplement. [file PPUL-60-0-s001.docx]

**Supplementary material: Adding early postnatal parameters of ventilation to prognostic models for pulmonary outcome in very preterm infants**

Birte Staude MD, Eva-Maria Mair, Maria Zernickel, Antje Westhoff, Rahel Schuler, Frank Oehmke MD, Harald Ehrhardt MD

**E-Table 1. Maternal and neonatal baseline characteristics by BPD28.**

**E-Table 2. Ventilatory parameters within the first 24 and 72 hours of life by BPD28.**

**E-Table 3. Model discrimination for BPD28.**

**E-Table 4.Full models for BPD28**

**E-Table 5. Full models for BPD36**

**E-Figure 1. Bland-Altman-Plots for assessing precision of calculation of mean airway pressure.**

**E-Figure 2. Calibration plots for BPD28 models in first 24 hours.**

**E-Figure 3. Calibration plots for BPD28 models in first 72 hours.**

**E-Figure 4. Calibration plots for BPD36 models in first 24 hours.**

**E-Figure 5. Calibration plots for BPD36 models in first 72 hours.**

**Supplementary information of definition of baseline characteristics.**

**E-Table 1. Maternal and neonatal baseline characteristics by BPD28.**

|  | **Overall** | **no BPD28** | **BPD28** | **p-value** |  |
| --- | --- | --- | --- | --- | --- |
| N (%) | 168 (100.0%) | 71 (42.3%) | 97 (57.7%) |  |  |
| **Neonatal baseline characteristics** | | | | |  |
| **Birthweight [g]** |  |  |  |  |  |
| Median [IQR] | 970.0[758.8, 1285.0] | 1300.0 [990.0, 1450.0] | 840.0 [700.0, 970.0] | <0.001 |  |
| **Gestation [weeks + days]** | |  |  |  |  |
| Median [IQR] | 28+1 [26+3, 30+0] | 30+0 [29+1, 30+5] | 26+6 [25+3, 28+0] | <0.001 |  |
| **Sex** |  |  |  |  |  |
| female | 92 (54.8%) | 39 (54.9%) | 53 (54.6%) | >0.999 |  |
| male | 76 (45.2%) | 32 (45.1%) | 44 (45.4%) |  |  |
| **Apgar 1‘** |  |  |  |  |  |
| Median [IQR] | 8.0 [7.0, 8.0] | 8.0 [7.0, 8.0] | 8.0 [7.0, 8.0] | 0.12 |  |
| **Apgar 5‘** |  |  |  |  |  |
| Median [IQR] | 9.0 [8.0, 9.0] | 9.0 [8.0, 9.0] | 9.0 [8.0, 9.0] | 0.06 |  |
| **Apgar 10‘** |  |  |  |  |  |
| Median [IQR] | 10.0 [9.0, 10.0] | 10.0 [9.0, 10.0] | 9.0 [9.0, 10.0] | 0.11 |  |
| **UA-pH** |  |  |  |  |  |
| Median [IQR] | 7.3 [7.2, 7.4] | 7.3 [7.2, 7.3] | 7.3 [7.2, 7.4] | 0.15 |  |
| Missing (%) | 22 (13.1%) | 6 (8.5%) | 16 (16.5%) |  |  |
| **X-ray within first 72 h - RDS grade** | | |  |  |  |
| 1 | 34 (20.2%) | 16 (22.5%) | 18 (18.6%) | 0.18 |  |
| 2 | 52 (31.0%) | 17 (23.9%) | 35 (36.1%) |  |  |
| 3 | 35 (20.8%) | 8 (11.3%) | 27 (27.8%) |  |  |
| 4 | 8 (4.8%) | 2 (2.8%) | 6 (6.2%) |  |  |
| Missing (%) | 39 (23.2%) | 28 (39.4%) | 11 (11.3%) |  |  |
| **Surfactant** |  |  |  |  |  |
| Yes | 107 (63.7%) | 28 (39.4%) | 79 (81.4%) | <0.001 |  |
| **Surfactant doses within 72h** | |  |  |  |  |
| Median [IQR] | 1.0 [0.0, 1.0] | 0.0 [0.0, 1.0] | 1.0 [1.0, 1.0] | <0.001 |  |
| **Maternal baseline characteristics** | | | | |  |
| **Maternal Age** |  |  |  |  |  |
| Median [IQR] | 31.0 [27.0, 36.0] | 32.0 [28.5, 36.0] | 30.0 [27.0, 35.0] | 0.43 |  |
| **Pregnancy** |  |  |  |  |  |
| Median [IQR] | 1.0 [1.0, 3.0] | 1.0 [1.0, 3.0] | 1.0 [1.0, 3.0] | 0.8 |  |
| **Parity** |  |  |  |  |  |
| Median [IQR] | 1.0 [1.0, 2.0] | 1.0 [1.0, 2.0] | 1.0 [1.0, 2.0] | 0.81 |  |
| **Mode of delivery** |  |  |  |  |  |
| C-section | 161 (95.8%) | 66 (93.0%) | 95 (97.9%) | 0.23 |  |
| Spontaneous | 7 (4.2%) | 5 (7.0%) | 2 (2.1%) |  |  |
| **Type of pregnancy** |  |  |  |  |  |
| singleton | 104 (61.9%) | 44 (62.0%) | 60 (61.9%) | >0.999 |  |
| multiple | 64 (38.1%) | 27 (38.0%) | 37 (38.1%) |  |  |
| **Nicotine** |  |  |  |  |  |
| yes | 26 (15.5%) | 13 (18.3%) | 13 (13.4%) | 0.53 |  |
| Missing (%) | 3 (1.8%) | 1 (1.4%) | 2 (2.1%) |  |  |
| **ANS** |  |  |  |  |  |
| none/ < 24h | 38 (22.6%) | 18 (25.4%) | 20 (20.6%) | 0.002 |  |
| > 24h/ < 7d | 86 (51.2%) | 26 (36.6%) | 60 (61.9%) |  |  |
| > 7d | 44 (26.2%) | 27 (38.0%) | 17 (17.5%) |  |  |
| **Cause for preterm delivery** | |  |  |  |  |
| AIS | 76 (45.2%) | 30 (42.3%) | 46 (47.4%) | 0.85 |  |
| Preeclampsia/HELLP | 33 (19.6%) | 16 (22.5%) | 17 (17.5%) |  |  |
| IUGR | 23 (13.7%) | 10 (14.1%) | 13 (13.4%) |  |  |
| Other | 36 (21.4%) | 15 (21.1%) | 21 (21.6%) |  |  |
| **Morbidities of prematurity** | | | | |  |
| **PDA** |  |  |  |  |  |
| no | 37 (22.0%) | 13 (18.3%) | 24 (24.7%) | 0.034 |  |
| PDA | 46 (27.4%) | 6 (8.5%) | 40 (41.2%) |  |  |
| Missing (%) | 85 (50.6%) | 52 (73.2%) | 33 (34.0%) |  |  |
| **PDA therapy (any)** |  |  |  |  |  |
| yes | 9 (5.4%) | 0 (0.0%) | 9 (9.2%) | 0.02 |  |
| **ROP** |  |  |  |  |  |
| no | 107 (63.7%) | 65 (91.5%) | 42 (43.3%) | <0.001 |  |
| yes | 61 (36.3%) | 6 (8.5%) | 55 (56.7%) |  |  |
| **ROP grade** |  |  |  |  |  |
| no ROP | 107 (63.7%) | 65 (91.5%) | 42 (43.3%) | <0.001 |  |
| 1 | 29 (17.3%) | 4 (5.6%) | 25 (25.8%) |  |  |
| 2 | 13 (7.7%) | 1 (1.4%) | 12 (12.4%) |  |  |
| ≥3 including APROP | 19 (11.3%) | 1 (1.4%) | 18 (18.6%) |  |  |
| **ROP therapy (any)** |  |  |  |  |  |
| ROP with therapy | 10 (6.0%) | 0 (0.0%) | 10 (10.3%) | 0.014 |  |
| **PVL** |  |  |  |  |  |
| PVL | 3 (1.8%) | 0 (0.0%) | 3 (3.1%) | 0.37 |  |
| **IVH** |  |  |  |  |  |
| IVH | 10 (6.0%) | 3 (4.2%) | 7 (7.2%) | 0.63 |  |
| **IVH grade** |  |  |  |  |  |
| 0 | 158 (94.0%) | 68 (95.8%) | 90 (92.8%) | 0.16 |  |
| 1 | 4 (2.4%) | 2 (2.8%) | 2 (2.1%) |  |  |
| 2 | 5 (3.0%) | 0 (0.0%) | 5 (5.2%) |  |  |
| 3 | 1 (0.6%) | 1 (1.4%) | 0 (0.0%) |  |  |
| 4 | 0 (0.0%) | 0 (0.0%) | 0 (0.0%) |  |  |
| **Pneumothorax** |  |  |  |  |  |
| yes | 3 (1.8%) | 1 (1.4%) | 2 (2.1%) | >0.999 |  |
| **Infection** |  |  |  |  |  |
| LOI | 21 (12.5%) | 2 (2.8%) | 19 (19.6%) | 0.003 |  |
| **Duration of invasive ventilation and non-invasive ventilatory support** | | | | |  |
| **Total time respiratory support (MV + NIV) [days]** | | | | |  |
| Median [IQR] | 33.0 [10.0, 54.0] | 8.0 [4.0, 17.5] | 50.0 [40.25, 65.75] | <0.001 |  |
| Missing (%) | 3 (1.8%) | 0 (0.0%) | 3 (3.1%) |  |  |
| **Invasive ventilation [days]** | |  |  |  |  |
| Median [IQR] | 0.0 [0.0, 1.0] | 0.0 [0.0, 0.0] | 0.0 [0.0, 2.0] | <0.001 |  |
| **BPD = bronchopulmonary dysplasia, UA-pH = umbilical artery pH, ANS = antenatal corticosteroids, PDA = patent ductus arteriosus, ROP = retinopathy of prematurity, PVL = periventricular leukomalacia, IVH = intraventricular haemorrhage, LOI = late onset infection** | | | | |  |
|  |  |  |  |  |  |
|  |  |  |  |  |  |

**E-Table 2. Ventilatory parameters within the first 24 and 72 hours of life by BPD28.**

|  | **Overall** | **no BPD** | **BPD** | **p-value** |  |
| --- | --- | --- | --- | --- | --- |
| N (%) | 168 (100.0%) | 71 (42.3%) | 97 (57.7%) |  |  |
| **Ventilation within first 24 h** | | | | |  |
| **Most invasive mode of respiratory support within 24 hours** | | | | <0.001 |  |
| HFNC | 3 (1.8%) | 3 (4.2%) | 0 (0.0%) |  |  |
| CPAP | 27 (16.1%) | 22 (31.0%) | 5 (5.2%) |  |  |
| NIPPV | 91 (54.2%) | 34 (47.9%) | 57 (58.8%) |  |  |
| SIPPV | 45 (26.8%) | 12 (16.9%) | 33 (34.0%) |  |  |
| HFO | 2 (1.2%) | 0 (0.0%) | 2 (2.1%) |  |  |
| **MAP_NIV_ (measured, not including calculated)** | | | |  |  |
| Median [IQR] | 8.7 [8.0, 9.2] | 8.7 [8.3, 9.2] | 8.6 [7.8, 9.2] | 0.6 |  |
| Missing   (%)* | 54 (59.3%) | 21 (61.8%) | 33 (57.9%) |  |  |
| **MAP_C-NIV_ (MAP_NIV_ including calculated + correction for NIV)†** | | | | |  |
| Median [IQR] | 6.1 [4.6, 7.0] | 5.4 [4.4, 6.6] | 6.6 [5.2, 7.4] | 0.002 |  |
| **PIP_MV_** |  |  |  |  |  |
| Median [IQR] | 16.0 [14.0, 20.0] | 15.5 [14.0, 17.0] | 16.0 [14.0, 20.0] | 0.87 |  |
| **PEEP_MV_** |  |  |  |  |  |
| Median [IQR] | 6.0 [5.4, 6.2] | 5.8 [5.0, 6.0] | 6.0 [5.8, 6.2] | 0.19 |  |
| **MAP_MV_** |  |  |  |  |  |
| Median [IQR] | 8.6 [7.6, 9.7] | 8.3 [7.9, 9.2] | 8.6 [7.5, 9.8] | 0.87 |  |
| **MAP_C-TP_ (including correction for NIV)†** | | | | |  |
| Median [IQR] | 6.7 [5.3, 7.8] | 6.0 [4.4, 7.1] | 7.3 [5.9, 8.3] | <0.001 |  |
| **Ventilation within first 72 h** | | | | |  |
| **Most invasive mode of respiratory support within 72 hours** | | | | <0.001 |  |
| HFNC | 3 (1.8%) | 3 (4.2%) | 0 (0.0%) |  |  |
| CPAP | 24 (14.3%) | 20 (28.2%) | 4 (4.1%) |  |  |
| NIPPV | 89 (53.0%) | 36 (50.7%) | 53 (54.6%) |  |  |
| IPPV | 49 (29.2%) | 12 (16.9%) | 37 (38.1%) |  |  |
| HFO | 3 (1.8%) | 0 (0.0%) | 3 (3.1%) |  |  |
| **MAP_NIV_ (measured, not including calculated)** | | | |  |  |
| Median [IQR] | 8.7 [8.0, 9.1] | 8.9 [8.5, 9.1] | 8.5 [7.7, 9.1] | 0.27 |  |
| Missing  (%)* | 54 (61.4%) | 23 (63.9%) | 31 (59.6%) |  |  |
| **MAP_C-NIV_ (MAP_NIV_ including calculated + corrected for NIV)** | | | | |  |
| Median [IQR] | 6.5 [5.3, 9.3] | 6.2 [4.4, 8.4] | 7.2 [5.7, 9.6] | 0.002 |  |
| **PIP_MV_** |  |  |  |  |  |
| Median [IQR] | 16.0 [15.0, 20.0] | 15.5 [14.0, 17.0] | 17.0 [15.0, 20.0] | 0.21 |  |
| **PEEP_MV_** |  |  |  |  |  |
| Median [IQR] | 6.0 [5.4, 6.3] | 5.8 [5.0, 6.0] | 6.0 [5.6, 6.4] | 0.13 |  |
| **MAP_MV_** |  |  |  |  |  |
| Median [IQR] | 8.7 [8.0, 9.1] | 8.9 [8.5, 9.1] | 8.5 [7.7, 9.1] | 0.27 |  |
| **MAP_C-TP_ (including correction for NIV)** | | | | |  |
| Median [IQR] | 7.7 [5.9, 9.4] | 6.5 [4.6, 9.0] | 8.4 [6.5, 10.0] | <0.001 |  |
| BPD = bronchopulmonary dysplasia, FiO_2_ = fraction of inspired oxygen, NIV = non-invasive ventilation, PIP = positive inspiratory pressure, PEEP = positive endexpiratory pressure, MAP = mean airway pressure, MV = mechanical ventilation, TP = total population. * Missing data calculated as a proportion of those receiving NIV. | | | | |  |
|  |  |  |  |  |  |
|  |  |  |  |  |  |
|  |  |  |  |  |  |
|  |  |  |  |  |  |

**E-Table 3. Model discrimination for BPD28.**

| **BPD28** | | | | | | | | |
| --- | --- | --- | --- | --- | --- | --- | --- | --- |
|  | AUC | Threshold | Specificity | Sensitivity | PPV | NPV | Accuracy | Calibration slope |
| Base model | 0.85 | 0.70 | 0.86 | 0.69 | 0.87 | 0.67 | 0.76 | 1.02 |
| **First 24 hours including:** | | | | | | | |  |
| FiO_2_ | 0.86 | 0.58 | 0.76 | 0.80 | 0.82 | 0.74 | 0.79 | 0.99 |
| MAP | 0.86 | 0.72 | 0.94 | 0.66 | 0.94 | 0.67 | 0.78 | 1.13 |
| RSS | 0.86 | 0.59 | 0.77 | 0.80 | 0.83 | 0.74 | 0.79 | 1.02 |
| **First 72 hours including:** | | | | | | | |  |
| FiO_2_ | 0.86 | 0.59 | 0.80 | 0.78 | 0.84 | 0.73 | 0.79 | 0.89 |
| MAP | 0.86 | 0.69 | 0.92 | 0.68 | 0.92 | 0.68 | 0.78 | 0.96 |
| RSS | 0.86 | 0.67 | 0.90 | 0.70 | 0.91 | 0.69 | 0.79 | 1.09 |
| Base model consists of birthweight, sex and antenatal corticosteroids. Full models are shown in E-Table 1 and 2. | | | | | | | | |

**E. Table 4. Full models for BPD28**

|  | **Estimate** | **SE** | **z-value** | **p-value** | **OR** | **95% CI lower** | **95% CI upper** |
| --- | --- | --- | --- | --- | --- | --- | --- |
| **Base model** | | | | | | | |
| Intercept | 4.42 | 0.89 | 4.94 | 0.000 | 83.12 | 14.41 | 479.40 |
| Birthweight (100 g) | -0.44 | 0.08 | -5.66 | 0.000 | 0.64 | 0.55 | 0.75 |
| Gender (male) | 0.65 | 0.40 | 1.62 | 0.106 | 1.92 | 0.87 | 4.22 |
| ANS (24 h - 7d) | 0.45 | 0.49 | 0.92 | 0.356 | 1.57 | 0.60 | 4.06 |
| ANS (> 7 d) | -0.07 | 0.53 | -0.13 | 0.901 | 0.94 | 0.33 | 2.67 |
| **FiO_2_: including max. FiO_2_ within first 24 hours of life** | | | | | | | |
| Intercept | 3.48 | 1.04 | 3.34 | 0.001 | 32.52 | 4.22 | 250.89 |
| Birthweight (100 g) | -0.45 | 0.08 | -5.56 | 0.000 | 0.64 | 0.55 | 0.75 |
| Gender (male) | 0.59 | 0.41 | 1.46 | 0.144 | 1.81 | 0.82 | 4.01 |
| ANS (24 h - 7d) | 0.51 | 0.49 | 1.03 | 0.303 | 1.66 | 0.63 | 4.34 |
| ANS (> 7 d) | -0.05 | 0.54 | -0.09 | 0.927 | 0.95 | 0.33 | 2.74 |
| max. FiO_2_ | 0.03 | 0.01 | 1.84 | 0.066 | 1.03 | 1.00 | 1.06 |
| **MAP: including max. MAP within first 24 hours of life** | | | | | | | |
| Intercept | 2.50 | 1.17 | 2.14 | 0.032 | 12.20 | 1.24 | 120.47 |
| Birthweight (100 g) | -0.38 | 0.08 | -4.78 | 0.000 | 0.68 | 0.58 | 0.80 |
| Gender (male) | 0.57 | 0.41 | 1.39 | 0.164 | 1.77 | 0.79 | 3.94 |
| ANS (24 h - 7d) | 0.54 | 0.50 | 1.09 | 0.277 | 1.71 | 0.65 | 4.53 |
| ANS (> 7 d) | 0.10 | 0.55 | 0.18 | 0.855 | 1.11 | 0.38 | 3.25 |
| max. MAP | 0.20 | 0.10 | 1.91 | 0.057 | 1.22 | 0.99 | 1.49 |
| **RSS: including max. RSS within first 24 hours of life** | | | | | | | |
| Intercept | 3.60 | 0.98 | 3.68 | 0.000 | 36.54 | 5.38 | 248.16 |
| Birthweight (100 g) | -0.43 | 0.08 | -5.33 | 0.000 | 0.65 | 0.56 | 0.76 |
| Gender (male) | 0.59 | 0.41 | 1.45 | 0.148 | 1.80 | 0.81 | 3.99 |
| ANS (24 h - 7d) | 0.55 | 0.49 | 1.11 | 0.268 | 1.73 | 0.66 | 4.56 |
| ANS (> 7 d) | 0.04 | 0.55 | 0.08 | 0.939 | 1.04 | 0.36 | 3.04 |
| max. RSS | 0.24 | 0.13 | 1.89 | 0.058 | 1.28 | 0.99 | 1.64 |
| **FiO_2_: including max. FiO_2_ within first 72 hours of life** | | | | | | | |
| Intercept | 3.19 | 1.04 | 3.06 | 0.002 | 24.29 | 3.15 | 187.22 |
| Birthweight (100 g) | -0.43 | 0.08 | -5.26 | 0.000 | 0.65 | 0.56 | 0.76 |
| Gender (male) | 0.58 | 0.41 | 1.43 | 0.153 | 1.79 | 0.81 | 3.97 |
| ANS (24 h - 7d) | 0.50 | 0.50 | 1.02 | 0.310 | 1.66 | 0.63 | 4.38 |
| ANS (> 7 d) | -0.01 | 0.54 | -0.01 | 0.989 | 0.99 | 0.34 | 2.89 |
| max. FiO_2_ | 0.03 | 0.01 | 2.15 | 0.031 | 1.03 | 1.00 | 1.05 |
| **MAP: including max. MAP within first 72 hours of life** | | | | | | | |
| Intercept | 2.22 | 1.13 | 1.98 | 0.048 | 9.23 | 1.02 | 83.73 |
| Birthweight (100 g) | -0.38 | 0.08 | -4.66 | 0.000 | 0.68 | 0.58 | 0.80 |
| Gender (male) | 0.48 | 0.41 | 1.16 | 0.247 | 1.61 | 0.72 | 3.63 |
| ANS (24 h - 7d) | 0.58 | 0.50 | 1.15 | 0.250 | 1.78 | 0.67 | 4.76 |
| ANS (> 7 d) | 0.14 | 0.55 | 0.25 | 0.804 | 1.15 | 0.39 | 3.39 |
| max. MAP | 0.21 | 0.09 | 2.32 | 0.020 | 1.24 | 1.03 | 1.48 |
| **RSS: including max. RSS within first 72 hours of life** | | | | | | | |
| Intercept | 3.14 | 0.99 | 3.18 | 0.001 | 23.17 | 3.34 | 160.61 |
| Birthweight (100 g) | -0.40 | 0.08 | -4.87 | 0.000 | 0.67 | 0.57 | 0.79 |
| Gender (male) | 0.51 | 0.41 | 1.23 | 0.218 | 1.66 | 0.74 | 3.70 |
| ANS (24 h - 7d) | 0.55 | 0.50 | 1.10 | 0.272 | 1.73 | 0.65 | 4.64 |
| ANS (> 7 d) | 0.10 | 0.55 | 0.18 | 0.857 | 1.10 | 0.37 | 3.27 |
| max. RSS | 0.28 | 0.12 | 2.39 | 0.017 | 1.33 | 1.05 | 1.68 |

Logistic regression models were built by bootstrapping the sample 1000 times and correcting shrinking the coefficients according to average optimism corrected slopes. Intercepts were recalculated afterwards. Max, maximum; ANS, antenatal corticosteroids; RSS, respiratory severity score.

**E-Table 5. Full models for BPD36**

|  | **Estimate** | **SE** | **z-value** | **p-value** | **OR** | **95% CI lower** | **95% CI upper** |
| --- | --- | --- | --- | --- | --- | --- | --- |
| **Base model** | | | | | | | |
| Intercept | 2.43 | 0.95 | 2.56 | 0.010 | 11.38 | 1.77 | 73.09 |
| Birthweight (100 g) | -0.46 | 0.10 | -4.45 | 0.000 | 0.63 | 0.52 | 0.77 |
| Gender (male) | 0.63 | 0.45 | 1.40 | 0.162 | 1.88 | 0.78 | 4.54 |
| ANS (24 h - 7d) | 0.17 | 0.54 | 0.31 | 0.754 | 1.19 | 0.41 | 3.44 |
| ANS (> 7 d) | -0.50 | 0.80 | -0.63 | 0.529 | 0.61 | 0.13 | 2.89 |
| **Model 1: including max. FiO_2_ within first 24 hours of life** | | | | | | | |
| Intercept | 0.85 | 1.15 | 0.74 | 0.462 | 2.33 | 0.24 | 22.19 |
| Birthweight (100 g) | -0.46 | 0.11 | -4.33 | 0.000 | 0.63 | 0.52 | 0.78 |
| Gender (male) | 0.69 | 0.47 | 1.48 | 0.140 | 2.00 | 0.80 | 5.00 |
| ANS (24 h - 7d) | 0.19 | 0.56 | 0.33 | 0.738 | 1.21 | 0.40 | 3.61 |
| ANS (> 7 d) | -0.66 | 0.81 | -0.81 | 0.416 | 0.52 | 0.11 | 2.53 |
| max. FiO_2_ | 0.04 | 0.01 | 2.95 | 0.003 | 1.04 | 1.01 | 1.07 |
| **Model 2: including max. MAP within first 24 hours of life** | | | | | | | |
| Intercept | -0.78 | 1.40 | -0.56 | 0.578 | 0.46 | 0.03 | 7.16 |
| Birthweight (100 g) | -0.40 | 0.11 | -3.77 | 0.000 | 0.67 | 0.54 | 0.83 |
| Gender (male) | 0.50 | 0.47 | 1.07 | 0.286 | 1.65 | 0.66 | 4.15 |
| ANS (24 h - 7d) | 0.34 | 0.57 | 0.60 | 0.549 | 1.41 | 0.46 | 4.33 |
| ANS (> 7 d) | -0.30 | 0.82 | -0.37 | 0.713 | 0.74 | 0.15 | 3.70 |
| max. MAP | 0.35 | 0.12 | 2.89 | 0.004 | 1.43 | 1.12 | 1.81 |
| **Model 3: including max. RSS within first 24 hours of life** | | | | | | | |
| Intercept | 1.33 | 1.08 | 1.24 | 0.216 | 3.78 | 0.46 | 31.16 |
| Birthweight (100 g) | -0.45 | 0.11 | -4.27 | 0.000 | 0.64 | 0.52 | 0.78 |
| Gender (male) | 0.62 | 0.47 | 1.32 | 0.188 | 1.86 | 0.74 | 4.69 |
| ANS (24 h - 7d) | 0.25 | 0.57 | 0.44 | 0.658 | 1.29 | 0.42 | 3.91 |
| ANS (> 7 d) | -0.50 | 0.81 | -0.62 | 0.536 | 0.60 | 0.12 | 2.98 |
| max. RSS | 0.36 | 0.12 | 3.06 | 0.002 | 1.43 | 1.14 | 1.80 |
| **Model 1: including max. FiO_2_ within first 72 hours of life** | | | | | | | |
| Intercept | 0.64 | 1.17 | 0.55 | 0.583 | 1.90 | 0.19 | 18.90 |
| Birthweight (100 g) | -0.44 | 0.10 | -4.19 | 0.000 | 0.65 | 0.53 | 0.79 |
| Gender (male) | 0.73 | 0.47 | 1.56 | 0.120 | 2.08 | 0.83 | 5.22 |
| ANS (24 h - 7d) | 0.32 | 0.57 | 0.55 | 0.580 | 1.37 | 0.45 | 4.24 |
| ANS (> 7 d) | -0.46 | 0.82 | -0.56 | 0.573 | 0.63 | 0.13 | 3.13 |
| max. FiO_2_ | 0.03 | 0.01 | 2.91 | 0.004 | 1.04 | 1.01 | 1.06 |
| **Model 2: including max. MAP within first 72 hours of life** | | | | | | | |
| Intercept | -0.50 | 1.37 | -0.37 | 0.713 | 0.61 | 0.04 | 8.80 |
| Birthweight (100 g) | -0.41 | 0.10 | -3.89 | 0.000 | 0.67 | 0.54 | 0.82 |
| Gender (male) | 0.44 | 0.47 | 0.94 | 0.347 | 1.56 | 0.62 | 3.92 |
| ANS (24 h - 7d) | 0.27 | 0.56 | 0.47 | 0.637 | 1.30 | 0.43 | 3.91 |
| ANS (> 7 d) | -0.32 | 0.81 | -0.40 | 0.689 | 0.72 | 0.15 | 3.52 |
| max. MAP | 0.30 | 0.11 | 2.80 | 0.005 | 1.35 | 1.09 | 1.66 |
| **Model 3: including max. RSS within first 72 hours of life** | | | | | | | |
| Intercept | 0.88 | 1.12 | 0.79 | 0.431 | 3.78 | 0.46 | 31.16 |
| Birthweight (100 g) | -0.42 | 0.10 | -4.02 | 0.000 | 0.64 | 0.52 | 0.78 |
| Gender (male) | 0.62 | 0.47 | 1.31 | 0.190 | 1.86 | 0.74 | 4.69 |
| ANS (24 h - 7d) | 0.36 | 0.58 | 0.61 | 0.543 | 1.29 | 0.42 | 3.91 |
| ANS (> 7 d) | -0.35 | 0.82 | -0.43 | 0.667 | 0.60 | 0.12 | 2.98 |
| max. RSS | 0.31 | 0.10 | 3.22 | 0.001 | 1.43 | 1.14 | 1.80 |

Logistic regression models were built by bootstrapping the ample 1000 times and correcting shrinking the coefficients according to average optimism corrected slopes. Intercepts were recalculated afterwards. Max, maximum; ANS, antenatal corticosteroids; RSS, respiratory severity score.

**
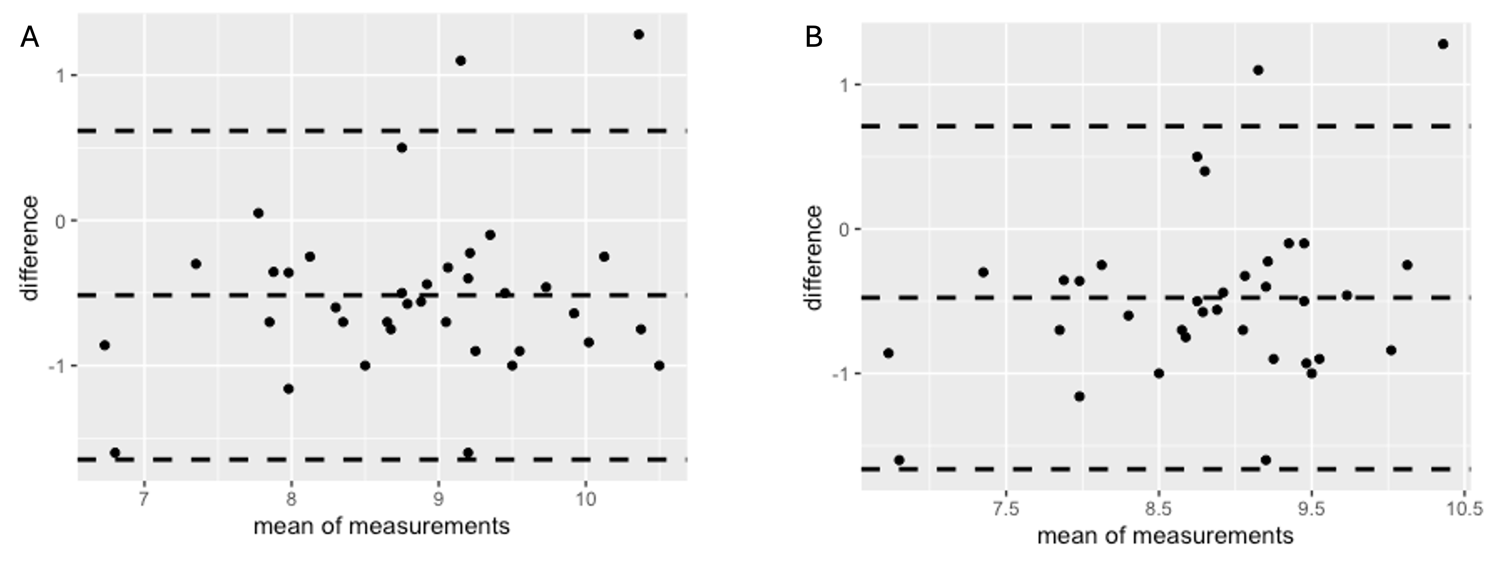
**

**E-Figure 1. Bland-Altman-Plots for assessing precision of calculation of mean airway pressure.**

1. Calculated mean airway pressure (MAP) in the first 24 hours of live compared to measured MAP for non-invasive positive pressure ventilation.
2. Calculated mean airway pressure (MAP) in the first 72 hours of live compared to measured MAP for non-invasive positive pressure ventilation.

**
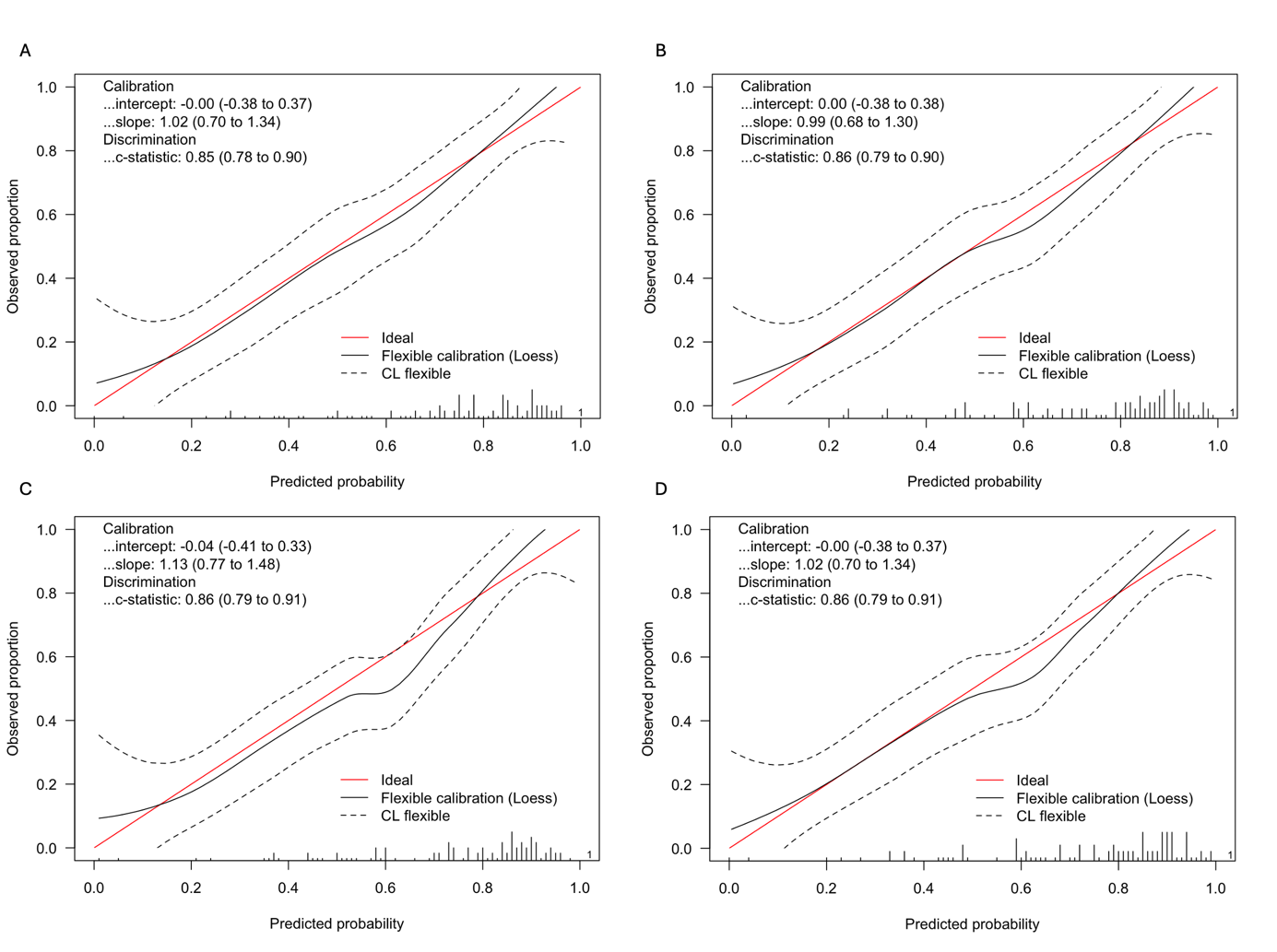
**

**E-Figure 2. Calibration plots for BPD28 models in first 24 hours.**

1. For the baseline model.
2. For the model including max. fraction of inspired oxygen within the first 24 hours.
3. For the model including max. mean airway pressure within the first 24 hours.
4. For the model including max. respiratory severity score within the first 24 hours.

**
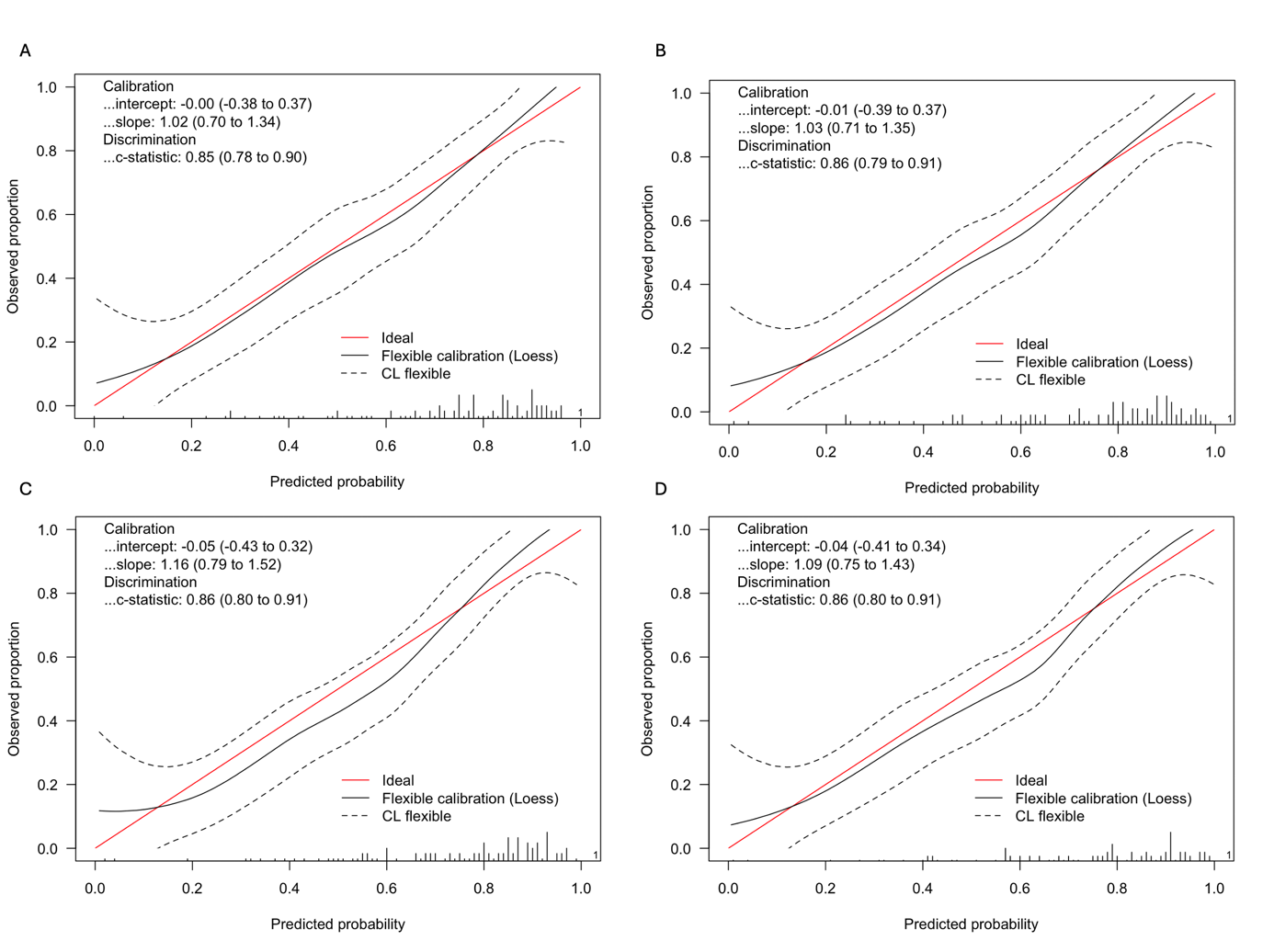
**

**E-Figure 3. Calibration plots for BPD28 models in first 72 hours.**

1. For the baseline model.
2. For the model including max. fraction of inspired oxygen within the first 72 hours.
3. For the model including max. mean airway pressure within the first 72 hours.
4. For the model including max. respiratory severity score within the first 72 hours.

**
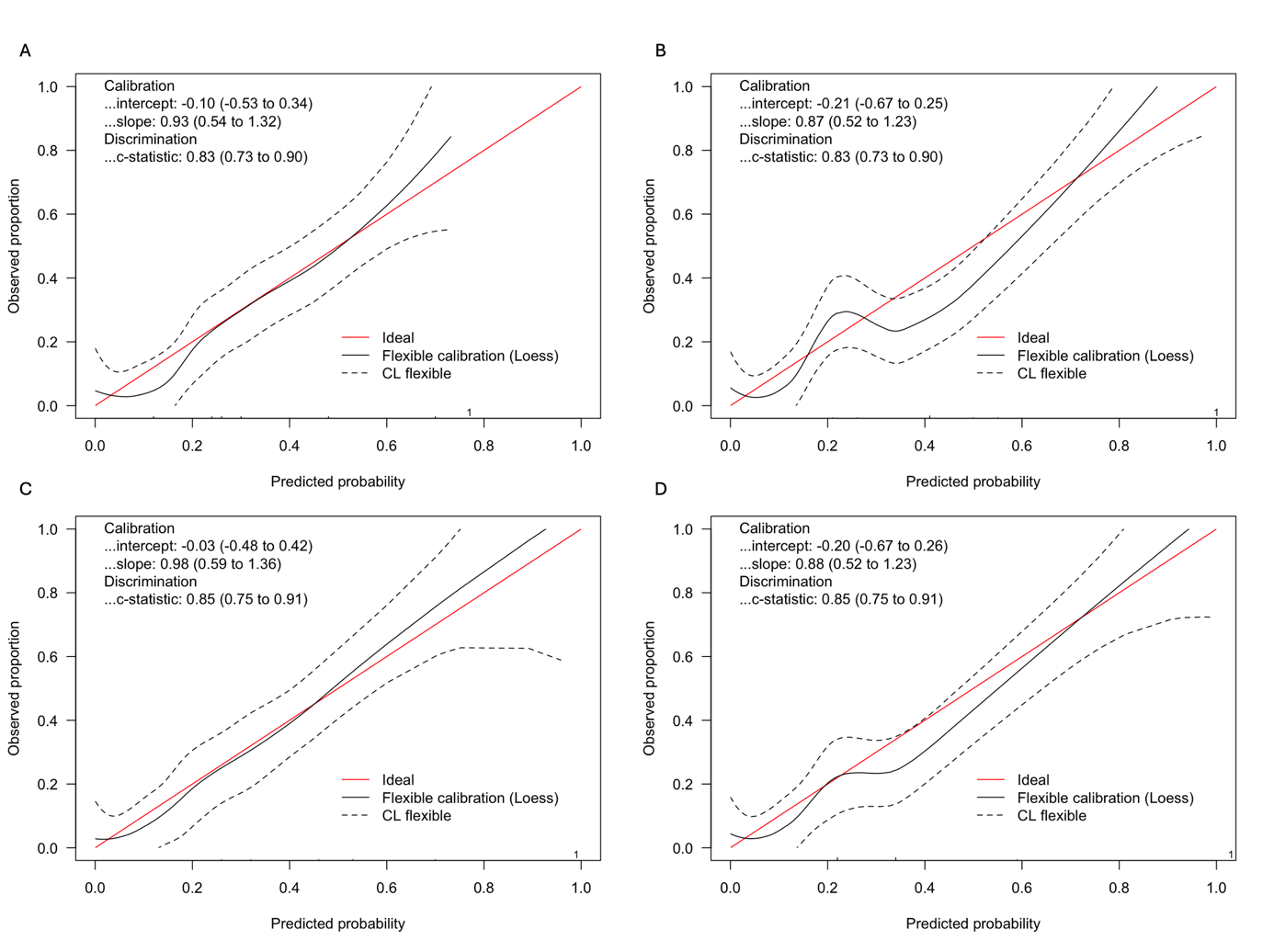
**

**E-Figure 4. Calibration plots for BPD36 models in first 24 hours.**

1. For the baseline model.
2. For the model including max. fraction of inspired oxygen within the first 24 hours.
3. For the model including max. mean airway pressure within the first 24 hours.
4. For the model including max. respiratory severity score within the first 24 hours.

**
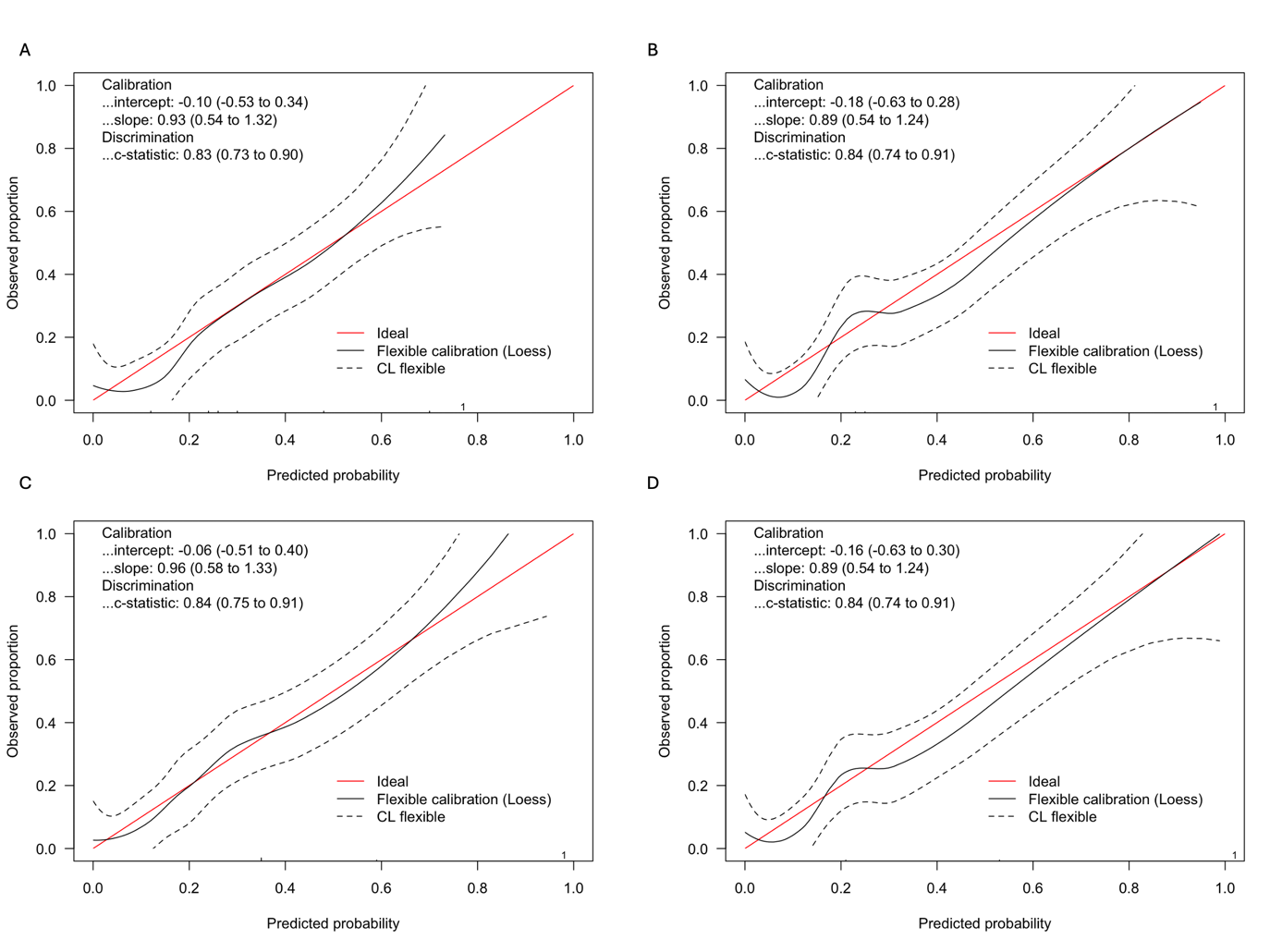
**

**E-Figure 5. Calibration plots for BPD36 models in first 72 hours.**

1. For the baseline model.
2. For the model including max. fraction of inspired oxygen within the first 72 hours.
3. For the model including max. mean airway pressure within the first 72 hours.
4. For the model including max. respiratory severity score within the first 72 hours.

**Supplementary information of definition of baseline characteristics.**

Maternal and neonatal baseline characteristics contained the following items: maternal age, number of pregnancy and parity, maternal nicotine consumption during pregnancy, type of pregnancy with birth as singleton or multiple, mode of delivery, cause of preterm delivery separated into intraamniotic infection, preeclampsia/eclampsia/HELLP, intrauterine growth restriction (IUGR) if noted as reason for delivery and other reasons. Delivery for intraamniotic infection fulfilled at least one of the following criteria as published recently: histopathologic chorioamnionitis, elevated AF interleukin-6 >3600pg/ml or delivery for intractable premature contractions under tocolytic therapy or for premature rupture of membranes (PPROM) preceding labour.

Neonatal baseline characteristics included gestational age, birth weight, sex, Apgar score at 1, 5 and 10 minutes of life and umbilical cord artery pH. We used the percentiles for weight, length, and head circumference of the German perinatal registry and counted small for gestational age (SGA) when birthweight was <10^th^ percentile. Neonatal acute severe outcomes included intraventricular hemorrhage (IVH), pneumothorax, late onset infection (LOI) applying the criteria from the German NICU nosocomial infection surveillance system (NEO-KISS), detection of a persistent ductus arteriosus (PDA) and PDA therapy, retinopathy of prematurity (ROP) and ROP therapy.

Respiratory characteristics included the severity of RDS according to the radiologic definition, the need and number of surfactant administrations irrespective of the mode of application and the decision for mechanical ventilation. The assessment of radiologic RDS severity was executed on the first available X-ray within 72 hours after birth independently by two trained NICU staff members blinded to the clinical course and differences in staging were jointly resolved^1^. Decision for intubation was done at the discretion of the attending neonatologist and was based on respiratory failure in the delivery room, FiO_2_ >40-60% after a maximum of three less invasive surfactant applications (LISA), pneumothorax, focal intestinal perforation or NEC ≥stage 2 and >1 severe prolonged apnea during standard dosage of caffeine. Respiratory support parameters comprised the need and duration of non-invasive ventilation (NIV) and mechanical ventilation (MV) counted in days. Positive end-expiratory pressure (PEEP), mean airway pressure (MAP) and peak inspiratory pressure (PIP) are detailed for NIV and MV.

**References**

1. Couchard M, Polge J, Bomsel F. [Hyaline membrane disease: diagnosis, radiologic surveillance, treatment and complications]. Annales De Radiologie. 1974;17(7):669–683.
